# An Integrated Multi‐Scale Approach to Habitat Modelling and Conservation for a Threatened Marsupial in a Dynamic and Fire‐Prone Landscape

**DOI:** 10.1002/ece3.72782

**Published:** 2025-12-25

**Authors:** Luke Lupone, Anthony Rendall, Raylene Cooke, Chloe Barker, John White

**Affiliations:** ^1^ School of Life and Environmental Sciences Deakin University Geelong Victoria Australia

**Keywords:** conservation planning, dynamic distributions, field validation, multi‐scale approach, southern brown bandicoot, species distribution models

## Abstract

We investigated the distribution and habitat associations of the southern brown bandicoot ( *Isoodon obesulus obesulus* ), a threatened marsupial, to understand how it occupies a dynamic, fire‐prone landscape in south‐east Australia. Our goal was to identify core habitat at landscape and local scales to inform conservation efforts. This study was conducted in Gariwerd (Grampians National Park), a fire‐prone landscape in south‐east Australia. We employed a multi‐faceted approach. First, we built and field‐validated Species Distribution Models (SDMs) to delineate landscape‐scale core habitat. To rigorously evaluate model performance and minimise bias, we conducted broad‐scale, systematic field surveys. Subsequently, we collected and analysed fine‐scale habitat characteristics at survey sites, using occupancy modelling to enhance our understanding of the species' ecological niche at a local scale. Our SDMs demonstrated good discriminative ability between presence and absence when tested on field data (AUC = 0.75). However, a notable proportion of observed absences occurred in high‐probability areas, leading to an overestimation of the species' distribution and negatively impacting overall evaluation metrics. This likely reflects the dynamic nature of bandicoot populations, introducing uncertainty into model predictions. Site‐level occupancy modelling revealed specific habitat associations: bandicoot occupancy was positively influenced by greater coverage of spear grass, slender twine‐rush, sedge, and the presence of diggings, while negatively associated with higher densities of *Callitris*. SDMs are valuable for demarcating core habitats for endangered species. However, to effectively inform conservation strategies for dynamic species like the southern brown bandicoot, model predictions must be rigorously validated with independent field surveys and complemented by fine‐scale habitat analyses. This multi‐scale approach is crucial for accurate conservation planning in dynamic landscapes.

## Graphical abstract

Our paper presents a novel, integrated, multi‐scale framework for habitat modelling and conservation planning. We demonstrate that for dynamic species, landscape‐scale models must be rigorously field‐validated and enriched by fine‐scale ecological data to provide actionable conservation guidance.

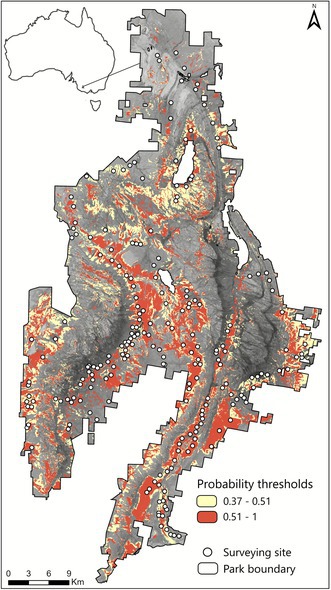

## 1 Introduction

Effective conservation of threatened species relies on understanding their distributions and ecological niches (Hirzel and Le Lay 2008; Wiens et al. 2010). Given accelerating biodiversity decline driven by climate change and human‐induced habitat loss, identifying and protecting core habitat has become an essential conservation strategy (Keppel et al. 2012; Mayani‐Parás et al. 2021). However, the rapid and widespread nature of these changes within landscapes presents significant challenges to effective conservation efforts (Butt et al. 2016; Camac et al. 2021; Pigot et al. 2023). Vulnerable species in dynamic landscapes, in particular, often exhibit shifting occupancy patterns, which can hinder the efficacy of conservation efforts (Greenville et al. 2016; Hale et al. 2016; Stobo‐Wilson et al. 2020). Therefore, investigating the habitat attributes influencing species' landscape and local‐scale distributions is critical for understanding their interactions within dynamic ecosystems. This knowledge is essential for developing climate‐adaptive conservation strategies that effectively address risks from climate‐driven disturbances such as fires and drought (Greenville et al. 2018; Hoffmann et al. 2019).

Australian marsupials are undergoing range shifts and declines due to climate‐driven disturbances and predation by introduced species such as the red fox ( *Vulpes vulpes* ) and feral cat ( *Felis catus* ) (hereafter foxes and cats) (Woinarski et al. 2015). Landscape‐scale processes, such as rainfall‐driven productivity, are key drivers of marsupial population dynamics and distributions; high rainfall often promotes increased reproduction, enhanced dispersal, and subsequent recolonisation of broader landscapes (Hale et al. 2016; Jones et al. 2023; White et al. 2022). Conversely, low‐rainfall periods and drought conditions can cause significant contractions in species distributions, as resource availability in suboptimal habitat becomes unsuitable (Hale et al. 2016; White et al. 2022).

During periods of contraction, species occupancy becomes more heavily influenced by local‐scale features such as persistent soil moisture and dense vegetation cover, which provide essential resources such as shelter and food (Pavey et al. 2017; Selwood and Zimmer 2020; Soderquist and Mac Nally 2000). Wildfire is a landscape‐scale process that alters habitat suitability, primarily by removing vegetation cover essential for many species' shelter and food resources (Chia et al. 2016; Kelly et al. 2010; Miritis et al. 2024). Post‐fire landscape regeneration depends on several local‐scale factors, including burn intensity and the productivity of recovering vegetation, which is influenced by terrain ruggedness (Bassett et al. 2017; Lindenmayer et al. 2021; Sharma et al. 2023). Consequently, fire‐prone landscapes typically support a diverse, dynamic mosaic of habitats in various successional stages, each offering suitable conditions for different groups of species over time (Fox 1982; Kelly et al. 2012). Projected increases in wildfire intensity and frequency, however, pose significant threats to ground‐dwelling marsupials, as widespread fires reduce habitat complexity and create ideal conditions for increased predation in post‐burn landscapes (Hradsky 2020). These changes present serious challenges for the effective management of these vulnerable species (Doherty et al. 2023).

While the responses of ground‐dwelling marsupials to drivers like rainfall and fire have been of significant research interest in Australia (Crowther et al. 2018; Greenville et al. 2016; Hale et al. 2016; Kelly et al. 2011), many insights still lack direct application to targeted landscape‐scale conservation efforts. Hence, there is a growing need to develop climate adaptation strategies that support threatened species' persistence against a backdrop of climatic fluctuations (Reside et al. 2019; Selwood et al. 2019). Investigating the occupancy patterns of threatened marsupials can provide insight into the source–sink dynamics shaping their distribution, thereby helping identify areas of greatest conservation value regardless of changing climate conditions. Critically, this requires examining habitat preferences across multiple spatial scales (Di Stefano et al. 2011; Dorph et al. 2021).

Species distribution modelling (SDM) is an increasingly valuable tool for predicting species distributions across a wide range of temporal and spatial scales (Kass et al. 2025). While many SDM approaches lack direct applicability to on‐ground conservation planning, models developed at finer landscape scales have proven valuable for guiding such efforts (Burns et al. 2020; Johnson et al. 2023; Thurtell et al. 2022). Despite their utility, however, numerous studies highlight the pitfalls and limitations of these approaches, particularly biases arising from unreliable presence or environmental data, or models' inability to fully capture the ecological processes driving species distributions (Araújo and Guisan 2006; Jarnevich et al. 2015; Lee‐Yaw et al. 2022). Such limitations are problematic for conservation planning, as inaccurate predictions of species distributions can undermine conservation efforts (Muscatello et al. 2021; Velazco et al. 2020).

Consequently, the collection of independent field data to validate models is repeatedly advocated as a crucial step in the modelling process (Cayuela et al. 2009; Johnson et al. 2023; Leroy et al. 2018). Importantly, this process can also facilitate the collection of additional site‐level habitat data, which can bridge broader‐scale application gaps and thereby enhance the description of core habitat for target species.

The southern brown bandicoot (eastern mainland subspecies; *Isoodon obesulus obesulus* (Shaw, 1797) Taxonomic Serial Number 709548) is an endangered species of ground‐dwelling marsupial endemic to Australia (Environment Protection and Biodiversity Conservation Act 1999 (Cth)). Southern brown bandicoots (hereafter *bandicoots*) are considered an ecologically important species, as their digging behaviour provides valuable ecosystem services (Fleming et al. 2014). These services include increased water infiltration, enhanced seed germination, and enhanced nutrient cycling through the dispersal of subterranean fungi (Elliott et al. 2023; Valentine et al. 2012, 2017).

The species has experienced substantial declines throughout its distribution due to habitat loss and predation from invasive cats and foxes (Coates et al. 2008; Coates and Wright 2003; Paull et al. 2013). Changing fire regimes have also contributed to the species' decline; evidence suggests that predation by cats and foxes is enhanced following fire, as bandicoots likely rely on dense understory vegetation for shelter (Arthur et al. 2012; Long 2009; Robley et al. 2016). Under climate change, with landscapes increasingly experiencing more frequent, larger, and more intense fires, bandicoots face a significantly elevated risk of landscape‐scale declines due to predation from introduced predators.

Previous research on bandicoots within the Grampians landscape of south‐east Australia indicates they are highly responsive to rainfall, exhibiting dynamic shifts in population size and distribution following extreme climatic events (Hale et al. 2016; Lupone et al. 2024). This response aligns with the concept of ‘boom‐and‐bust’ population dynamics (Letnic and Dickman 2006), where the species may become virtually undetectable during extended drought periods, yet responds rapidly following periods of high rainfall. This boom‐and‐bust dynamic suggests that viable populations persist within the landscape during bust periods, albeit at levels that often fall below detection thresholds during dry and post‐fire conditions.

This study employed multiple methods across different scales to locate and describe the core habitat of bandicoots in a highly dynamic and fire‐prone landscape. Specifically, we aimed to build and field‐validate a species distribution model (SDM) that uses thresholds to broadly delineate the species' core habitat at a landscape scale. To ensure comprehensive coverage of varying habitat suitability and adequate species representation, we conducted broad‐scale, systematic surveys across the study landscape. This approach minimised biases and improved the evaluation of our model's predictive performance. We then collected local‐scale habitat characteristics data to investigate attributes supporting bandicoot occupancy and to further enhance our understanding of the species' ecological niche.

## 2 Methods

### 2.1 Study Region

Gariwerd (Grampians National Park) is an important conservation reserve covering 168,241 ha in south‐east Australia. The landscape encompasses a series of sandstone mountain ranges with temperate woodlands and heathlands on their slopes and valley floors (Parks Victoria 2003). The broad vegetation types that cover much of the park are characterised by low, shrubby vegetation and nutrient‐poor soils; these systems are shaped by frequent fire events, which influence patterns of regeneration, species composition, and habitat structure (Cheal 2010; Enright et al. 2012; Keith et al. 2002). Sand Heathland and Heathy Woodland (Parks Victoria 2003) are the two dominant ecological vegetation classes (EVCs) in the park. Sand heathland is generally a treeless heath occurring in deep, infertile sands with occasional scattered emergent *Eucalyptus* trees and a diverse, dense heathy shrub layer made up of *Leptospermum, Banksia, Epacris, Grevillea* and an understory of grasses, rushes and sedges (DEECA 2021a). Heathy Woodland is a low woodland with a *Eucalyptus*‐dominated canopy cover (15%) with a dense middle story of heathy shrub layer made up of *Leptospermum, Banksia, Epacris, Grevillea*, and an understory of grasses, rushes and sedges (DEECA 2021a).

The region has a 50‐year average annual rainfall of 718 mm, an average maximum temperature of 21 (°C) in the hottest month of the year (January), and an average minimum temperature of 0.9 (°C) in the coolest month of the year (July) (Bureau of Meteorology 2023). A recent history of extreme climatic events has contributed to an elevated fire history within the park, with 90% of the area having burned in three major wildfire events between 2006 and 2014 (Lupone et al. 2024). Prescribed burning has also been increased within the park as a tool to lower fuel loads and prevent future large‐scale wildfires (Parks Victoria 2003). Consequently, more areas within the landscape are in more frequent post‐fire and early stages of regeneration, which is documented as contributing to increased predation of ground‐dwelling marsupials by exotic predators (Hradsky 2020).

### 2.2 Landscape‐Scale Distribution Modelling (SDM)

#### 2.2.1 Occurrence Records of Bandicoot in the Landscape

We used the Grampians National Park boundary as a calibration area for the study. Almost all records of southern brown bandicoot in this region are from within the Grampians National Park, and as such this region constitutes our inference domain. Occurrence records for the southern brown bandicoot within this boundary were compiled from multiple sources, yielding an initial dataset of 226 observations from Parks Victoria and the Victorian Biodiversity Atlas (VBA) spanning 2003–2023 (DEECA 2021b). To address potential spatial bias arising from inclusion of clustered records in our models (multiple records in very close proximity), we implemented a spatial filtering process. This thinning was implemented using a 90‐m minimum‐distance criterion by retaining a single record from neighbouring occurrences and removing the others (Steen et al. 2021). This 90 m threshold was deemed appropriate given the 30 m × 30 m cell size of our environmental layers, which helped ensure a degree of spatial independence between the final 119 presence records (once clustered records were thinned) used in subsequent species distribution modelling.

#### 2.2.2 Background Samples

Presence‐only species distribution modelling requires the use of background samples or “pseudo‐absences” instead of true absence data (Phillips et al. 2009). While pseudo‐absences are often randomly selected across the region of interest, various methods exist to refine their selection and potentially improve final model performance (Barbet‐Massin et al. 2012; Chapman et al. 2019; Vollering et al. 2019). To assess potential sampling bias in our presence data, we visually examined the spatial distribution of all survey records within the park (Lupone et al. 2026). Given the extensive and geographically diverse nature of surveys in the landscape, which were not limited by habitat or elevation, we opted not to adjust background selection specifically to account for sampling bias in the presence dataset distribution.

Acknowledging, however, that background samples should reflect the study region's geography and critically the species' biology (Chapman et al. 2019; Jarnevich et al. 2017), we strategically manipulated the selection of 10,000 background points. Specifically, we increased the sampling density at lower elevations within the park using a bias file generated from a kernel density estimation applied to a Digital Elevation Model (DEM). This approach deliberately down‐weighted the selection of background samples from higher elevations, which in this landscape are characterised by expansive rocky outcrops where the southern brown bandicoot is not known to occur (Coates et al. 2008; Paull et al. 2013; Robinson et al. 2018). By preferentially sampling background points from the woodland slopes and heath habitats at lower elevations, we aimed to improve model discrimination of suitable habitat and gain a more accurate understanding of the species' ecological niche, rather than artificially inflating model performance by including unsuitable environments (Jarnevich et al. 2017; Lobo et al. 2008).

#### 2.2.3 Environmental Data

All environmental layers were processed using ARC GIS 10.14 (ESRI 2014), and all data were resampled to a cell size of 30 m × 30 m (Table 1). As vegetation is known to influence occupancy of our target species (Paull et al. 2013), multiple layers measuring vegetation features were incorporated. Ecological Vegetation Classes (EVCs) are groupings of vegetation communities based on floristics and structure (DEECA 2021a). These classes are further grouped to provide a broader classification of vegetation types, termed EVC groups, which were included as a categorical variable in the modelling (hereafter EVC groups).

**TABLE 1:** Environmental variables (and sources) used in Maxent modelling of southern brown bandicoot distribution in the Gariwerd landscape.

Variable layer	Description	Data source
NDVI	Normalised Vegetation Difference Index measured and averaged over six measurements during record breaking drought episode	United States Geological Surveys (USGS) GloVis Landsat
Rain (mm)	Average annual rainfall in the region	ANUCLIM Version 6.1. Fenner School of Environment and Society at the Australian National University
Time since fire (years)	A shapefile of burn history containing time since fire in years	Department of Energy, Environment and Climate Action (DEECA), Fire history overlay of most recent fires, https://discover.data.vic.gov.au/
Slope (°)	The slope derived from the DEM‐H	Calculated using ArcGIS with GeoScience Australia 1 Second SRTM Digital Elevation Model (DEM) https://www.ga.gov.au/
Watercourse (m)	Distance to water courses from occurrence data	Department of Energy, Environment and Climate Action (DEECA), Vicmap Hydro—Watercourse Line, https://discover.data.vic.gov.au/
EVC groups (categorical)	A shapefile containing generalised subgroups of dominant Ecological Vegetation Classes in the Grampians national park	Department of Energy, Environment and Climate Action (DEECA), Native Vegetation—Modelled 2005 Ecological Vegetation Classes (with Bioregional Conservation Status), https://discover.data.vic.gov.au/

Normalised Difference Vegetation Index (NDVI) was included as a measure of vegetation productivity. NDVI is derived from satellite imagery (United States Geological Surveys (USGS) GloVis Landsat, 30 m resolution) and measures the ‘greenness’ of environments (Berry et al. 2007; Coops et al. 2013) scaled between −1 and +1, with lower values representing less productive vegetation and higher scores indicating ‘greener’ and more densely vegetated (Lanorte et al. 2014). Specifically in this study, our NDVI layer was built by averaging six annual measurements from the park, taken during the driest period of the year (peak Austral summer, Jan–March) prior to the 2006 wildfire. This period represents the latter end of Australia's “Millennium Drought”, when the landscape was under extreme water stress (Van Dijk et al. 2013). This extreme drought‐based NDVI metric has been demonstrated as a good predictor of drought refugia in this landscape (White et al. 2022). High values for this extreme drought‐based NDVI layer align very well with areas, such as gullies and drainage lines, that retain significant soil moisture and as such have considerable dense vegetation, a critical habitat element for many small terrestrial mammal species and previously shown to influence mammal occupancy in this landscape (Hale et al. 2016).

Time since fire was calculated from annual fire mapping records of the park (DEECA 2024). This variable is dynamic, as each presence record was linked to its corresponding time since fire (years). Despite the extensive fire history in the park, the dataset we used includes substantial pre‐fire records and spans a broad range of fire‐age classes, which is important for capturing early post‐fire habitat dynamics. In heathland and heathy woodland, the most pronounced structural and compositional changes typically occur within the first decade after fire (Cheal 2010). Average annual rainfall was sourced from ANUCLIM (Xu and Hutchinson 2013). Slope was calculated in ArcGIS using Shuttle Radar Topography Mission Digital Elevation Model (SRTM DEM) (Geoscience Australia 2011). Distance to water course was calculated in ArcGIS using governmental watercourse data (DEECA 2024). Multicollinearity between environmental variables was assessed, with no variables being highly correlated (|*r*| < 0.7).

#### 2.2.4 Model Building and Evaluation

Candidate models were built using the ENMeval package (Kass et al. 2021), which performs automated tuning and evaluation of selected Maxent model settings to determine optimal model fit and predictive ability. We generated an initial candidate set of 20 models for bandicoot utilising all combinations of Linear (L) and quadratic (Q) features across a range of regularisation multipliers (RM) from 0 to 5 in increments of 0.5. Hinge, product, and threshold features were excluded from models to generate more ecologically realistic response curves and avoid overfitting of data (Merow et al. 2013).

The maxent algorithm produces two commonly applied metrics for evaluating variable importance: percentage contribution and permutation importance; here we prioritise permutation importance (Elith et al. 2006). Model selection was determined by two evaluation metrics calculated from discrimination (sensitivity and specificity): area under the receiver operating characteristic curve (AUC) and 10% training omission rate (OR_10_) (Fielding and Bell 1997; Kass et al. 2021). AUC‐ROC is a widely used, threshold‐independent metric for evaluating SDM performance, measuring accuracy across all possible thresholds, with scores > 0.7 having good discrimination and scores near 0.5 suggesting performance no better than random (Fielding and Bell 1997). OR_10_ is a threshold‐dependent metric that identifies the model with the lowest omission rate (false negatives) when the threshold is set to omit no more than 10% of the training presence records with the lowest predicted suitability (Kass et al. 2021). The best supported model was subsequently selected based on having the lowest average OR_10_ and the highest AUC.

The best supported models for bandicoot distribution were predicted against environmental data using the ‘cloglog’ output format to produce a relative likelihood of occurrence from 0 to 1 (Phillips et al. 2017). Predictive performance of this model was then evaluated on independent field data using multiple thresholds and their derived metrics, as well as threshold‐independent and calibration metrics (Phillips and Elith 2010).

### 2.3 Field Validation of SDM

#### 2.3.1 Site Selection for Field Surveys

Our predicted probability of occurrence SDM raster for bandicoot was used as the basis for systematic surveying of our study landscape. To avoid biasing evaluation metrics, our survey was designed to achieve comprehensive coverage across all predicted probabilities of occurrence. We achieved this by using the threshold that maximised sensitivity and specificity (MAXSS) to guide the selection of surveying sites. Probabilities were divided into three bins: the first encompassing areas with a probability below the MAXSS threshold, delineating unsuitable habitat. The remaining two bins were created by splitting the range of probabilities above MAXSS into two equal halves, enabling sampling of areas deemed moderately suitable and highly suitable. A total of 214 survey sites were then stratified across these three probability of occurrence categories. While recognising that designation of thresholds is inherently arbitrary in ecological terms, this method of systematic surveying facilitated robust coverage across a range of probabilities and was deemed appropriate to enhance validity of discrimination and calibration evaluation metrics.

During the field season (Jun‐Aug) of 2024, 214 sites were surveyed using a single motion‐sensing camera trap (Browning trail camera BTC‐6FHD). Camera traps were mounted on nearby trees where possible or on stakes at approximately 0.30 m from the ground and pointed at lures positioned 1–1.5 m in front of the camera trap and approximately 0.10 m off the ground. Lures consisted of 10 cm long, highly perforated PVC tubes filled with Holofill (soaked in an attractant mixture consisting of peanut butter, tuna oil, linseed oil and vanilla extract) secured to the ground by a metal peg and sprinkled with a cup of rolled oats (*sensu* White et al. 2022; Lupone et al. 2026). Camera traps were set to take a series of three images over 2 s once the camera was triggered by motion. The camera trap then had a dormant period of 30 s during which time it would not be triggered again. This substantially limits repeat triggering of the camera and saves both disk space and battery life. Each camera trap was deployed for a minimum of 14 nights with an average of 24 nights. Sites that did not achieve this 14‐night minimum were excluded from evaluation metrics unless a bandicoot presence was recorded during the actual deployment period. However, all sites, including those with shorter deployments, were used in subsequent occupancy modelling approaches.

Once images were downloaded from camera traps they were initially passed through the ‘camtrapR’ r package to assign time and date stamps to each image (Niedballa et al. 2016). Images were then individually checked with the aim of documenting each detection of a bandicoot. At each site this generated a detection/non‐detection sequence for each night of trap deployment. This data formed the basis of data for occupancy modelling in the ‘unmarked’ package in R version 4.1.2 (Fiske and Chandler 2015; R Core Team 2023).

#### 2.3.2 Using Field Data to Evaluate the SDM

To evaluate SDM predictions against independent field data, we used both threshold‐independent (AUC‐ROC, COR, calibration plots) and threshold‐dependent (sensitivity, specificity, Kappa, TSS) methods. We calculated AUC‐ROC scores using predicted probabilities of occurrence and observed site presence‐absence. To provide a rank‐free and threshold‐independent estimate of model performance, we calculated COR, a measure of calibration between model predictions and observations, ranging from 0 to 1 (Elith et al. 2006). Calibration plots were also used to visually assess the agreement between predicted probabilities and observed presence‐absences (Phillips and Elith 2010). These metrics quantify how closely specific predicted values align with observations, thereby providing an indication of the ‘goodness of fit’ of the model predictions.

Threshold‐dependent metrics for model performance were derived from confusion matrices generated using two thresholds: the threshold that maximised specificity/sensitivity (MAXSS) determined during model training, and an ‘Optimal’ threshold determined *post hoc* by identifying the MAXSS threshold for independent field data (Liu et al. 2013). The foundational metrics from confusion matrices are sensitivity (the proportion of observed presences that are predicted correctly) and specificity (the proportion of observed absences that are predicted correctly) (Fielding and Bell 1997; Pearce and Ferrier 2000). Other threshold‐based metrics used to assess model prediction accuracy included the Kappa statistic and True Skill Statistic (TSS), both of which evaluate performance relative to random chance. These metrics range from −1 to +1, with +1 indicating perfect prediction and values of zero or below indicating performance no better than random (Allouche et al. 2006). Both metrics have been shown to be negatively impacted by prevalence; however, we prioritise TSS over Kappa as a more robust measure of model performance when dealing with low prevalence datasets (Allouche et al. 2006; Lawson et al. 2014; Leroy et al. 2018).

### 2.4 Local‐Scale Habitat Attribute Modelling

#### 2.4.1 Site Level Habitat Characteristics

To measure habitat characteristics at a finer scale, each camera trap site was surveyed for local habitat features. Vegetation surveys used 10 m radius plots centred on the camera. Vegetation structure was measured using a 180 cm structure pole, divided into 10 cm increments, held vertically. At each site, we recorded the number of plant touches within each increment (to a maximum of 10 touches in each increment). These measurements were taken at four equidistant points around the plot and one at the centre, totalling five measurements per site. These values at each increment were averaged and multiplied by 10 to produce a percentage cover. Increments were then averaged over multiple height ranges to give percentage cover at 0–20 cm, 20–50 cm, 50–100 cm, 100–140 cm and 140–180 cm.

Vegetation types (taxonomic genus) common within Grampians habitats and deemed potentially important indicators of habitat type were then measured as percentage cover within the 10 m radius at each plot. Understory tree and shrub vegetation types included: *Leptospermum* sp., *Banksia* spp., *Melaleuca* spp., *Hakea* spp., *Allocasuarina* spp., *Xanthorrhoea* spp., *Grevillea* spp., *Acacia* spp., *Callitris* spp. Ground cover vegetation types included *Spear grass* (*Austrostipa* sp.), *Tassel rope‐rush* (*Hypolaena* sp.), *Slender twine‐rush* (*Leptocarpus* sp.), *Button‐grass* (*Gymnoschoenus* sp.). Species identified as *sedges* were pooled into one group but mostly comprised the genera *Gahnia* spp. and *Lepidosperma* spp. Logs were also measured as percentage coverage within the 10 m radius with a log defined as any woody material with a diameter > 5 cm. Within each 10 m radius plot, we also recorded the number of conical‐shaped diggings (Digs) that we suspected to be bandicoot in origin, based on their characteristic size and form (Valentine et al. 2012). These diggings were counted during each survey to provide a measure of bandicoot activity at the sites.

#### 2.4.2 Occupancy Modelling

Occupancy models were employed to assess the influence of site‐level variables on bandicoot occupancy. Detection probability was held constant across all sites and surveys, under the assumption that survey conditions were standardised (e.g., consistent lure and deployment protocols). Environmental site‐level variables, in contrast, were included to model variation in site occupancy. Predictor variables for occupancy probability included the site‐specific predicted probability of occurrence derived from species distribution models (*PPO*), vegetation characteristics (floristic composition and structural), and indicators of bandicoot activity (*Digs*). We developed a large suite of potential candidate occupancy models informed by both ecological reasoning and observed statistical relationships among covariates. Model selection was based on Akaike's Information Criterion corrected for small sample sizes (AICc), with models exhibiting a ΔAICc < 2 considered to have substantial support (Anderson and Burnham 2002). All analyses were conducted using the ‘unmarked’ package in R version 4.1.2 (Fiske and Chandler 2015; R Core Team 2023).

## 3 Results

### 3.1 Species Distribution Model Based on Historical Locations

**FIGURE 1:**
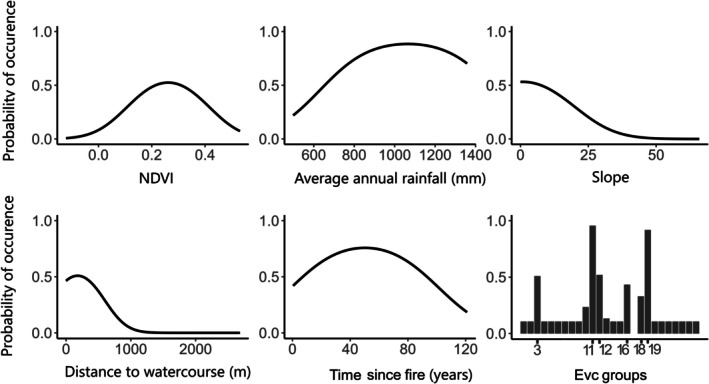
Relative likelihood of occurrence of southern brown bandicoot in response to environmental variables used in modelling. For the EVC groups shown, the categories represent: (3) Heathy woodlands (Dry and/or better drained), (11) Heathlands (sandy/well drained) (12) Riparian Scrubs or Swampy Scrubs and Woodlands, (16) Lower Slopes or Hills Woodlands (Herb‐rich), (18) Lowland forests, (19) Heathlands (not well drained).

**FIGURE 2:**
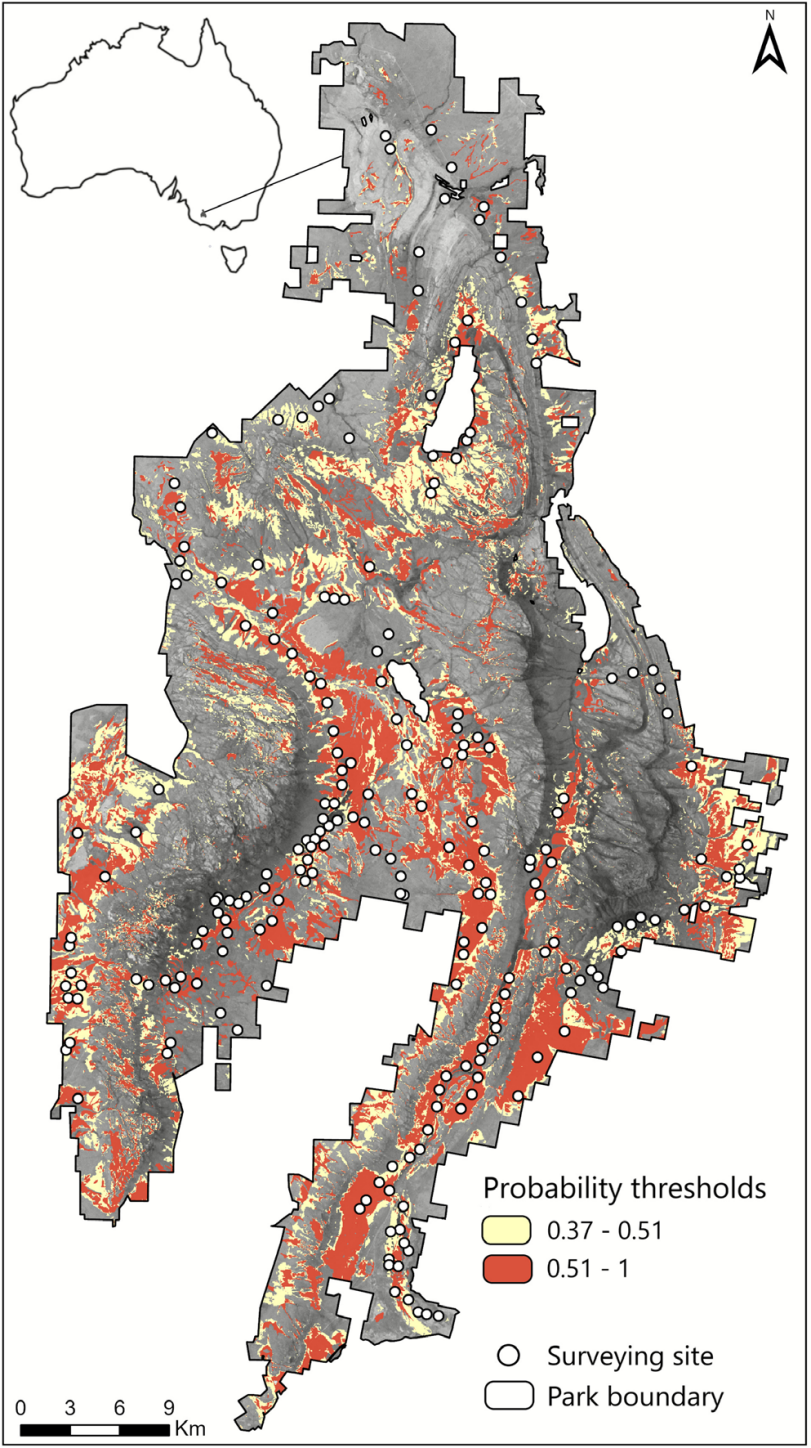
Species distribution model for southern brown bandicoot in the Gariwerd landscape. Shading indicates the relative likelihood of occurrence (0–1). Pink shading represents habitat above the threshold that maximised sum of sensitivity and specificity (MAXSS) on withheld testing data during model evaluation; areas below this threshold remain unshaded. Red shading highlights habitat above the ‘optimal’ threshold that maximised the sum of sensitivity and specificity but was determined using independent field data.

The best‐supported model for the bandicoot had an AUC of 0.83 and the lowest omission rate of 0.152 when compared to other candidate models. Ecological Vegetation Class (EVC) group was the most influential variable based on both permutation importance (PI = 57.5%) and percentage contribution (PC = 69.3%). Bandicoot occurrence was highest in Heathlands (both sandy/well‐drained and not well drained) compared to all other EVC groups. Occurrence was also relatively high in Riparian Scrubs or Swampy Scrubs, Heathy Woodlands (dry and/or better drained), Lower Slopes or Hills Woodlands (herb‐rich), and Lowland Forests (Figure 1). Distance to watercourse and slope (PI = 13.8% and 12.9%, respectively) displayed negative relationships, indicating the species prefers flatter habitats at the base of slopes in closer proximity to water courses. Rain, NDVI and time since fire were the least important variables (PI = 9.2%, 5.1%, 1.6%, respectively) and displayed unimodal responses to probability of bandicoot occurrence (Figure 1). The threshold value that maximised specificity/sensitivity (MAXSS) for model predictions during model evaluation was determined to be 0.37, and this value was subsequently used to generate threshold‐dependent metrics for evaluation against independent field data (Figure 2; Table 2).

**TABLE 2:** Evaluation metrics for cross‐validation and field validation of southern brown bandicoot distribution model.

***Threshold dependent***		***MAXSS***		***Optimal***	
		**0.37**		**0.514**	
**Confusion matrix**					
Observed (y)		Presence (y)	Absence (y)	Presence (y)	Absence (y)
Predicted (ŷ)	Presence (ŷ)	60	83	58	66
	Absence (ŷ)	4	55	6	72
Kappa		0.252		0.342	
True positive rate/*sensitivity* (TPR)		0.938		0.906	
True negative rate/*specificity* (TNR)		0.399		0.522	
False positive rate (FPR)		0.601		0.478	
False negative rate (FNR)		0.063		0.094	
Fale omission rate (FOR)		0.068		0.077	
True skill statistic (TSS)		0.336		0.428	
Proportion correctly classified (PCC)		0.569		0.644	
Misclassification rate (MCR)		0.431		0.356	
***Threshold independent***		***AUC***_***ROC***_		***COR***	
Model evaluation (Cross‐validation)		0.83		0.155	
Field validation (Independent data)		0.75		0.413	

*Note:* Multiple threshold‐dependent confusion matrices (shaded cells correctly predicted presences and absences, non‐shaded cells incorrectly predicted presences and absences) and derived evaluation metrics for presence‐absence independent field data. Thresholds used are maximum sum of sensitivity and specificity (MAXSS) and (optimal) threshold that represent maximum sensitivity and specificity calculated for field data. Threshold‐independent evaluation metrics from initial model cross‐validation and evaluation of independent field data.

### 3.2 Camera Trapping Surveys

A total of 5183 survey nights were conducted across 214 sites, producing 279,243 images. Of these, 202 (94%) sites met the minimum deployment of 14 nights, which allowed us to field‐validate the SDM with high confidence (a 14‐night deployment provides a 94.8% probability of detection given a nightly detection rate of 0.19), and these sites were retained for the evaluation metrics. Across these 202 sites, southern brown bandicoots were recorded at 64 (32%) locations. All 214 sites, however, were included in the subsequent occupancy modelling, as the framework explicitly accounts for imperfect detection; while shorter deployments may increase the chance of false negatives, any remaining non‐detections would be expected to make habitat–occupancy relationships more conservative rather than generating spurious effects.

### 3.3 Evaluation of Species Distribution Model Based on Field Data

The model achieved an AUC‐ROC value of 0.75 when evaluated against independent field data, indicating good discriminatory ability between occupied and unoccupied sites (Table 2). It demonstrated high sensitivity, with a true positive rate (TPR) of 0.938, meaning approximately 94% of all presences were correctly classified. Conversely, the model showed lower specificity, with a true negative rate (TNR) of 0.399, indicating that a higher proportion of observed absences were incorrectly predicted as presences. Kappa and TSS scores of 0.252 and 0.336, respectively, indicated weak to moderate agreement between predicted and observed presence‐absence at survey sites.

**FIGURE 3:**
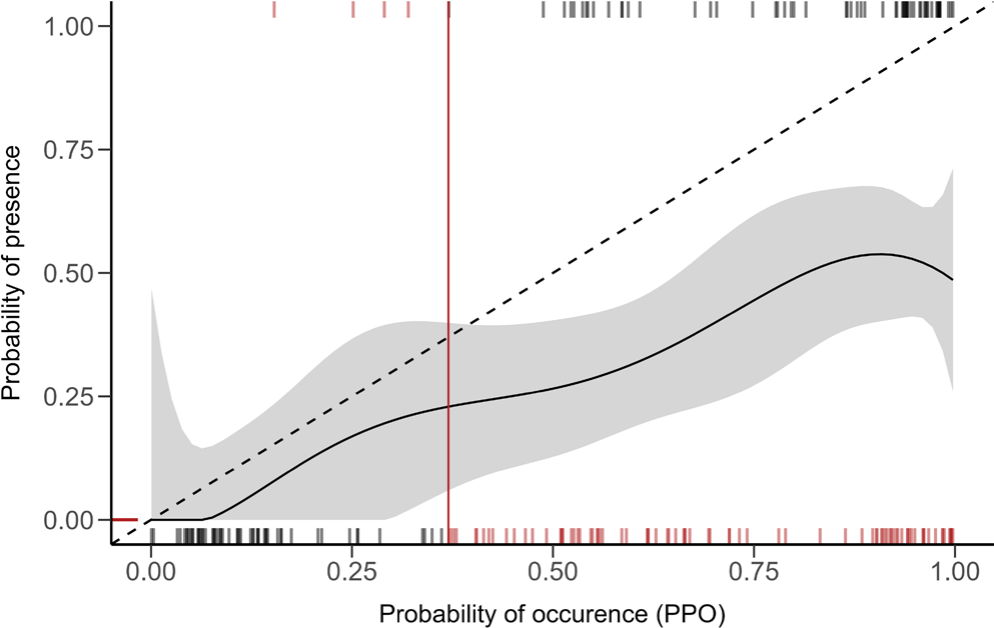
Presence‐absence calibration plot with regression line (Loess) measuring relationship between predicted probability and observed presence‐absence at sites. The shaded area represents the 95% confidence interval around the regression line. A diagonal‐dashed line indicates perfect agreement between predictions and observed presence‐absence. Rug plots show predicted value for presences (top rug) and absences (bottom rug). The vertical line (red) indicates the ‘MAXSS’ threshold that maximised the sum of sensitivity and specificity for model predictions on independent field data. False presences and false absences are indicated by red ticks (bottom and top rug respectively). True presences and true absences are indicated by black ticks (bottom and top rugs respectively).

The ‘optimal’ threshold generated *post hoc* by maximising the sum of sensitivity and specificity on our independent field data (optimal: 0.514) produced improved metrics (Kappa: 0.342, TSS: 0.468). These scores indicate a better ability to discriminate between presence and absence at sites (Table 2). Our model scored a lower AUC‐ROC when tested on independent field data compared to the training data (Table 2). However, model predictions showed higher calibration with independent field data than with withheld testing data (COR = 0.413, 0.155, respectively). Calibration plots visually illustrate the distribution of field presence/absence across the gradient of predicted probability of occurrence from SDMs, with a higher proportion of presences occurring in high probability areas. This is further supported by the positive relationship observed between site occupancy and predicted probability of presence (Figure 3).

### 3.4 Occupancy Modelling of Site Level Characteristics

**FIGURE 4:**
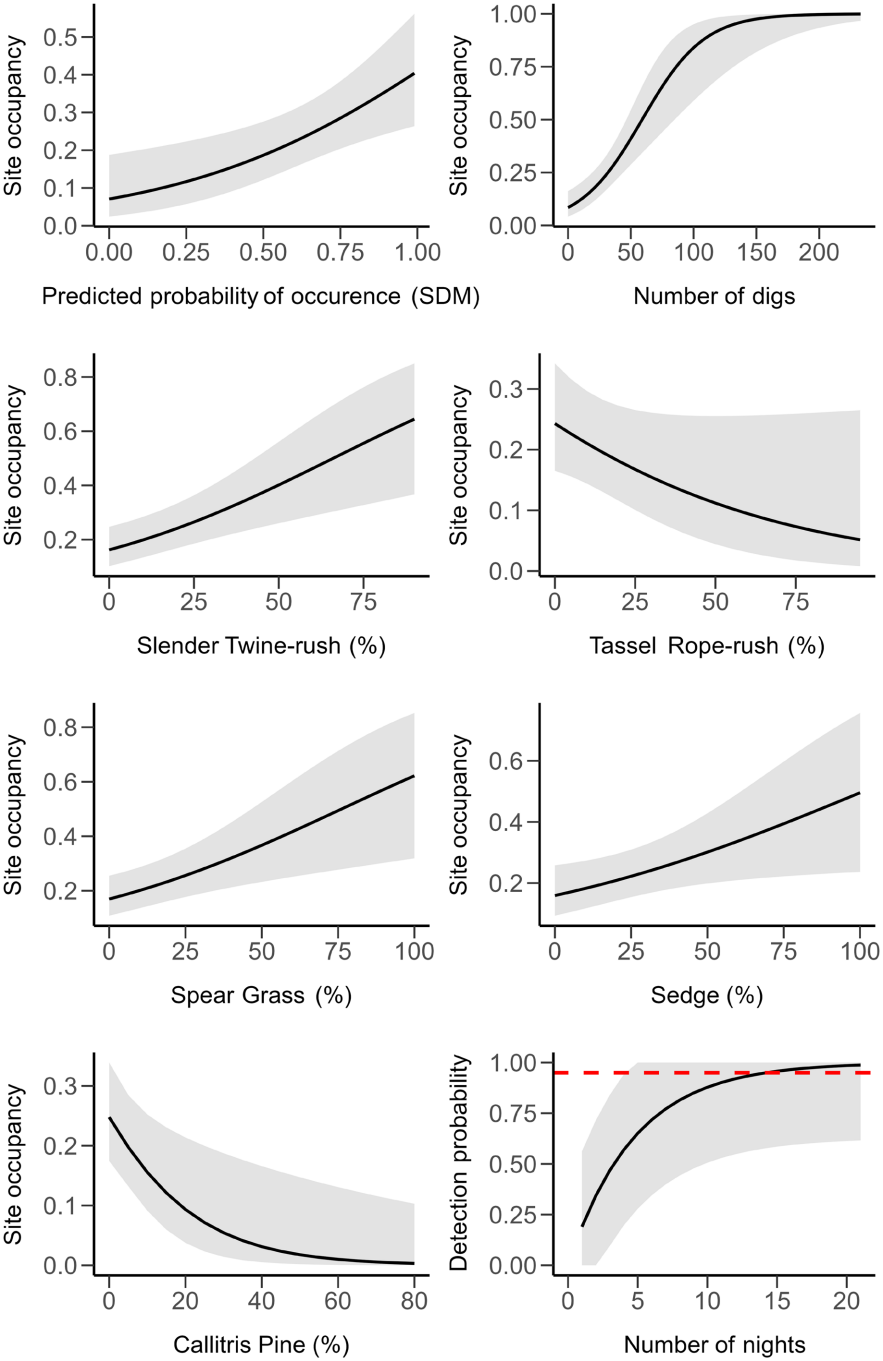
Site occupancy and detection probability of southern brown bandicoots at survey sites across the Gariwerd landscape. The first seven panels show predicted relationships (±95% confidence intervals) between site occupancy and the key influential predictor variables derived from the top‐ranked occupancy model. The final panel shows the cumulative detection probability over increasing survey nights (red dashed line represents the 95% detection threshold).

Bandicoots had a nightly detection probability of 0.19, resulting in a 94.8% probability of detecting the species at sites with our 14 nights of sampling (Figure 4). The most supported model for bandicoot site occupancy (AICc weight = 0.057; Table 3) included the following covariates: *Callitris*, Diggings (*Digs*), Slender Twine‐rush (*Leptocarpus*), Predicted Probability of Occurrence (*PPO*), *Sedge, Tassel Rope‐rush* (*Hypolaena*), and *Spear Grass* (*Austrostipa*). Importantly, six of these covariates, *Callitris*, Digs, *Slender Twine‐rush*, *PPO*, *Sedge*, and *Spear Grass*, occurred in every model within the ΔAICc < 2 set, indicating consistent support across all top‐supported models. Support also existed for *Button grass* (∆AICc = 0.12), *Leptospermum* (∆AICc = 0.47), *and* Vegetation density at 0–20 cm (∆AICc = 1.56; Table 3).

**TABLE 3:** Top ranking models for site occupancy and detection probability of southern brown bandicoot showing number of parameters (K), model log‐likelihood (LL), Akaike's Information Criterion corrected for small sample sizes (AICc), delta AIC (∆AICc), Akiake weights (AICc ω) and explanatory power of the models (*R* ^2^).

Model	K	LL	AICc	∆AICc	AICc ω	*R* ^2^
ѱ(CPine + Digs + ST‐rush +PPO + Sedge +TR‐rush +SprG)p(.)	9	−861.691	1742.3	0.00	0.057	0.40
ѱ(BtnG + CPine + Digs + ST‐rush + PPO + Sedge + TR‐rush + SprG)p(.)	10	−860.648	1742.4	0.12	0.054	0.40
ѱ(BtnG + CPine + Digs + Lepto + ST‐rush + PPO + Sedge + TR‐rush + SprG)p(.)	11	−859.713	1742.7	0.47	0.045	0.41
ѱ(CPine + Digs + Lepto + ST‐rush + PPO + Sedge + TR‐rush + SprG)p(.)	10	−860.997	1743.1	0.82	0.038	0.40
ѱ(BtnG + CPine + Digs + ST‐rush + PPO + Sedge + SprG)p(.)	9	−862.318	1743.5	1.26	0.031	0.39
ѱ(CPine + Digs + ST‐rush + PPO + Sedge + SprG)p(.)	8	−863.411	1743.5	1.26	0.031	0.39
ѱ(BtnG + CPine + Digs + Lepto + ST‐rush + PPO + Sedge + SprG)p(.)	10	−861.255	1743.6	1.33	0.029	0.40
ѱ(BtnG + CPine + Digs + ST‐rush + PPO + Sedge + SprG +0‐20 cm)p(.)	10	−861.368	1743.8	1.56	0.026	0.40
ѱ(CPine + Digs + ST‐rush + PPO + Sedge + SprG +0‐20 cm)p(.)	9	−862.478	1743.8	1.57	0.026	0.39
ѱ(CPine + Digs + ST‐rush + PPO + Sedge + TR‐rush + SprG +0‐20 cm)p(.)	10	−861.387	1743.9	1.60	0.026	0.40
ѱ(BtnG + CPine + Digs + ST‐rush + PPO + Sedge + TR‐rush + SprG +0‐20 cm)p(.)	11	−860.319	1744.0	1.68	0.025	0.41
ѱ(BtnG + CPine + Digs + Lepto + ST‐rush + PPO + Sedge + SprG +0‐20 cm)p(.)	11	−860.323	1744.0	1.69	0.025	0.41
ѱ(CPine + Digs + Lepto + ST‐rush + PPO + Sedge + SprG)p(.)	9	−862.613	1744.1	1.84	0.023	0.39

*Note:* Only models with an ΔAICc < 2 are included. Variables include *Callitris Pine* (CPine), *Digs* (Digs), *Slender Twine‐rush* (ST‐rush), *Predicted Probability of Occurrence* (PPO), *Sedge* (Sedge), *Tassel Rope‐rush* (TR‐rush), *Spear Grass* (SprG), *Button grass* (BtnG), *Leptospermum* (Lepto), Vegetation density at 0–20 cm (0–20 cm).

Regarding the influence of these covariates on occupancy, bandicoot occupancy was significantly negatively associated with higher densities of *Callitris* (β = −0.058, 95% CI = −0.103 to −0.013). Conversely, occupancy was significantly positively associated with greater coverage of *Spear Grass* (β = 0.021, 95% CI = 0.007 to 0.035), *Slender Twine‐rush* (β = 0.025, 95% CI = 0.010 to 0.040), and *Sedge* (β = 0.017, 95% CI = 0.002 to 0.031). Dig counts (*Digs*) were also significant and positively influenced site occupancy, with higher dig counts reflecting a higher probability of site occupancy (β = 0.040, 95% CI = 0.022 to 0.058; Figure 4).

Other variables were included in the top‐supported models (i.e., those with ∆AICc < 2), but their relationships with occupancy were not statistically significant (as their 95% CIs crossed zero). These included Tassel Rope‐rush (β = −0.019, 95% CI = −0.040 to 0.002), Vegetation density at 0‐20 cm (β = −0.208, 95% CI = −0.510 to 0.094), Button grass (*Gymnoschoenus*) (β = 0.018, 95% CI = −0.007 to 0.044), and *Leptospermum* (β = 0.009, 95% CI = −0.006 to 0.025) (Table 3).

Models with ΔAICc < 2 had *R* ^2^ values ranging from 0.39 to 0.41, indicating similar levels of explanatory power among top‐ranked models (Table 3).

## 4 Discussion

We outline a multi‐scale, multi‐method approach for identifying and describing core habitat for a threatened species in a dynamic landscape. By integrating landscape‐scale environmental data into species distribution models, and validating predictions using independent field surveys, we were able to assess both the discrimination and calibration while identifying the broad habitat patterns associated with bandicoot occurrence. As expected, discriminatory performance declined slightly when evaluated with independent data, while calibration improved, a pattern consistent with the tendency for pseudo‐absence–based internal validation to appear overly optimistic (Newbold et al. 2010; Jiménez‐Valverde 2020). Overall, the model provides a reliable yet precautionary approximation of habitat suitability, with thresholds offering a generous but defensible representation of bandicoot distribution. This broader approach is strengthened by integrating fine‐scale habitat assessments, which complement the regional model and support more informed, on‐ground conservation decision‐making aimed at long‐term species persistence.

### 4.1 Model Evaluation and Limitations

Species Distribution Models, projected at a fine‐scale and incorporating biologically relevant spatial data, can effectively estimate landscape‐scale distributions of bandicoots. We report a wide variety of evaluation metrics that offer varying measures of predictive performance and therefore need to be prioritised in the context of the study aims (Sofaer et al. 2019). As a threshold‐independent metric, AUC‐ROC indicated good discriminatory ability (> 0.7) (Fielding and Bell 1997), although discrimination alone does not assess model fit (Valavi et al. 2022). Calibration plots provided a more informative assessment, showing survey effort across the probability gradient and a positive relationship between predicted suitability and observed presences. However, they also revealed a high number of false positives within the MAXSS‐defined suitable range, leading to reduced predictive accuracy in the highest probability quartile and influencing threshold‐dependent metrics. This illustrates how selecting a single threshold from continuous predictions can be inherently arbitrary, with presence–absence classifications sensitive to where the threshold is placed. Binary maps are often more useful for conservation planning because they clearly identify priority areas for management (Jiménez‐Valverde 2014; Liu et al. 2013; Tulloch et al. 2016). The strong influence of EVC groups, which form discrete, mappable habitat units, supports the use of thresholds that convert continuous predictions into practical conservation layers for bandicoot in this landscape.

Our results demonstrate that threshold selection requires a compromise between enhancing sensitivity (proportion of observed presences correctly predicted) or specificity (proportion of observed absences correctly predicted), with prioritisation of one reducing the other (Liu et al. 2013). All reported evaluation metrics were negatively impacted by the high number of false positives (i.e., sites predicted as presences yet unoccupied) in our dataset. Despite a potential tendency to overpredict highly suitable habitat, our chosen thresholds demonstrated high sensitivity, accurately classifying nearly all known presences and a substantial proportion of absences. We consider our chosen thresholds to be reliable, as they reflect a precautionary approach that prioritises lower omission rates while tolerating higher false positives. This represents an intentional and desirable bias for threatened‐species modelling, as it reduces the risk of overlooking core habitat and ensures that potentially important areas are not excluded from conservation efforts.

The relatively low prevalence of bandicoot in our field dataset highlights a significant challenge in distribution modelling for endangered species. A high number of false positives introduces uncertainty in model interpretation, making it difficult to determine whether this reflects overprediction or genuinely low prevalence (Jiménez‐Valverde et al. 2013; Velazco et al. 2020). Numerous ecological factors can contribute to the overprediction of habitat suitability, as models inevitably fail to capture the complex and often interacting abiotic and biotic processes driving the dynamic and non‐uniform distribution of bandicoots (Jarnevich et al. 2015; Lee‐Yaw et al. 2022). For example, predation, a key biotic interaction, is often not captured by broad‐scale modelling but can significantly influence bandicoot presence, even in otherwise suitable habitat (Scheele et al. 2017).

These limitations are further amplified by the dynamic nature of this landscape, which is considered fire‐prone and experiences frequent wildfires and highly variable rainfall. Research of these processes in this landscape suggests they can drive rapid habitat transitions that result in site‐level colonisations and extinctions of bandicoots that are hypervariable through time (Hale et al. 2016; Lupone et al. 2024; White et al. 2022). During prolonged dry periods, with higher frequencies of fires and a greater impact of predation by invasive species, declines in bandicoots are often observed, likely leading to their contraction into more favourable areas. In contrast, during wetter periods following high rainfall years, individuals will reproduce and disperse into areas that during drier periods would be considered unsuitable (Lupone et al. 2024; White et al. 2022). While our models incorporated a dynamic time since fire variable and an NDVI metric intended to represent underlying gradients of soil moisture and vegetation productivity, they inherently fail to fully capture fine‐scale spatiotemporal patterns of this nature (Sinclair et al. 2010). Likewise, fine‐scale occupancy models are also influenced by unmeasured behavioural and environmental processes operating at temporal and spatial scales finer than the covariates included, meaning that patterns of occurrence may partially reflect these underlying dynamics rather than solely the measured habitat attributes. Such limitations are inherent in species habitat modelling approaches and highlight the risk of relying solely on withheld testing data to evaluate model performance (Jarnevich et al. 2015; Leroy et al. 2018). Independent field data should instead be used to validate and refine models, as well as to guide field surveys that generate complementary data to support this process (Feeley and Silman 2011).

### 4.2 Landscape and Local Occupancy of Bandicoot

Broad vegetation type is a dominant driver of bandicoot occupancy, with a preference for heathland and adjoining heathy woodland and riparian habitats (Paull et al. 2013; Robinson et al. 2018). These habitats often comprise dense ground cover and are typically situated at the bottom of slopes and along drainage lines (DEECA 2021a). Bandicoots are documented inhabiting a diverse range of habitats across their distribution; however, dense vegetation is a recurring variable (Claridge et al. 2019; Haby et al. 2013; Hope 2012; Maclagan et al. 2020; Robinson et al. 2018; Stoddart and Braithwaite 1979). The position of these heath and riparian habitats likely allows them to collect water runoff and sustain higher levels of vegetation growth than surrounding habitats (Groves and Specht 1965; Selwood et al. 2015; White et al. 2022). These habitats provide critical resources such as dense vegetation for food, nesting, and protection from invasive predators (Arthur et al. 2012; Haby et al. 2013; MacGregor et al. 2023; Zylinski et al. 2022). There is also evidence bandicoots will use multiple habitat types within their home ranges, including foraging in more open woodland areas that offer diverse foraging resources (Haby et al. 2013); hence, EVC groups such as heathy or lowland slope woodlands and forests also exhibited higher predicted occupancy. Dense heath habitats likely serve as core habitat for bandicoots, used for nesting and shelter, while they forage in the adjacent slope woodland and lowland forests. This is supported by our local‐scale analysis, which indicates that bandicoot site occupancy is primarily influenced by greater coverage of dense ground‐layer vegetation, such as sedge, slender twine‐rush, button grass, and spear grass. Additionally, bandicoot occupancy was negatively associated with the presence of *Callitris*, a species more commonly found on rocky outcrops and sparsely vegetated hillsides (DEECA 2021a).

Our modelling approach also provides insight into how bandicoot respond to major disturbances such as fire, although these relationships can be complex and context‐dependent. Time since fire was not a strong predictor of bandicoot occupancy in Gariwerd, aligning with the mixed conclusions regarding the influence of fire history on bandicoot occupancy. Some studies show increasing occupancy with time since fire (Arthur et al. 2012; Claridge and Barry 2000; Long 2009), while others find weak or even negative relationships (Catling et al. 2001; Lupone et al. 2024; Rees et al. 2024; Swan et al. 2021). This variation likely reflects local factors such as remnant refugia, fire intensity (Long 2009; MacGregor et al. 2023) and post‐fire predation pressure that shape bandicoot survival and recolonisation (Hope 2012; Hradsky 2020; Robley et al. 2016). Given our single‐season data and limited replication across fire‐age classes, we were unable to fully resolve these spatiotemporal dynamics. Nevertheless, previous work shows that bandicoots can persist or return to partially burnt habitats within a few years of fire (Hope 2012; Paull 1993; Smith 2013), particularly where rapid regrowth of sedges and grasses can quickly re‐establish adequate ground cover (Groves and Specht 1965).

### 4.3 Management Implications

Understanding the habitat preferences and core habitat for bandicoots is essential for effective management in Gariwerd. This understanding of habitat suitability can inform landscape‐scale strategies through the identification of high conservation priority areas. Such insights can in turn guide more targeted feral predator control and support fire management planning. Strategically protecting high‐priority heathlands and implementing longer‐term monitoring of these regions will likely facilitate bandicoot persistence.

Bandicoots exhibit ‘boom and bust’ population dynamics, suggesting these core habitats also function as climate refugia. During dry ‘bust’ periods (Letnic and Dickman 2010; Pavey et al. 2014), these core habitats likely support the persistence of the species and serve as source populations during wetter ‘boom’ periods, facilitating dispersal across the landscape (Brandle and Moseby 1999; Dickman et al. 2011). The ability to spatially identify these core habitats is therefore essential in climate adaptation planning, as these areas are critical to supporting metapopulation dynamics across drought‐ and fire‐prone landscapes (Morelli et al. 2017; Reside et al. 2019; Selwood et al. 2019; Selwood and Zimmer 2020).

## 5 Conclusions

Tracking the distributions of highly threatened species across ever‐changing landscapes presents a significant challenge. Successfully addressing this requires an understanding of the factors influencing species distributions at both landscape and local scales. This study employed multiple approaches to deepen our understanding of a threatened species' ecological niche within a fire‐prone environment. We demonstrated the practical application of species distribution models (SDMs) that incorporate biologically relevant, landscape‐scale environmental data to identify core habitat for endangered species. Our field validation of SDMs provided a crucial opportunity not only to test model accuracy but also to support the collection of additional site‐level data to further inform our understanding of species' habitat associations. This, in turn, enhanced our understanding of fine‐scale habitat preferences. Such knowledge is essential for effective conservation planning, particularly in the face of future climate extremes such as drought and bushfire.

## Author Contributions

**Luke Lupone:** conceptualization (lead), data curation (lead), formal analysis (lead), investigation (lead), writing – original draft (lead), writing – review and editing (equal). **Anthony Rendall:** conceptualization (equal), formal analysis (equal), supervision (equal), visualization (equal), writing – original draft (supporting), writing – review and editing (supporting). **Raylene Cooke:** conceptualization (equal), funding acquisition (supporting), investigation (supporting), project administration (equal), supervision (equal), writing – original draft (supporting), writing – review and editing (supporting). **Chloe Barker:** formal analysis (supporting), investigation (equal), writing – original draft (supporting), writing – review and editing (supporting). **John White:** conceptualization (equal), formal analysis (supporting), funding acquisition (lead), investigation (supporting), methodology (equal), project administration (equal), supervision (equal), writing – original draft (supporting), writing – review and editing (supporting).

## Funding

This work was supported by Parks Victoria.

## Conflicts of Interest

The authors declare no conflicts of interest.

## Data Availability

The data that support the findings of this study are openly available in Zenodo at https://zenodo.org/records/17706443.
